# Synchronized Drumming Enhances Activity in the Caudate and Facilitates Prosocial Commitment - If the Rhythm Comes Easily

**DOI:** 10.1371/journal.pone.0027272

**Published:** 2011-11-16

**Authors:** Idil Kokal, Annerose Engel, Sebastian Kirschner, Christian Keysers

**Affiliations:** 1 Department of Neuroscience, University Medical Center Groningen, Groningen, The Netherlands; 2 Donders Institute for Brain, Cognition and Behaviour, Centre for Cognitive Neuroimaging, Radboud University Nijmegen, Nijmegen, The Netherlands; 3 Max Planck Institute for Psycholinguistics, Nijmegen, The Netherlands; 4 Music Cognition & Action Group, Max Planck Institute for Human Cognitive and Brain Sciences, Leipzig, Germany; 5 Department of Developmental and Comparative Psychology, Max Planck Institute for Evolutionary Anthropology, Leipzig, Germany; 6 The Netherlands Institute for Neuroscience, Royal Netherlands Academy of Arts and Sciences (KNAW), Amsterdam, The Netherlands; University of Bologna, Italy

## Abstract

Why does chanting, drumming or dancing together make people feel united? Here we investigate the neural mechanisms underlying interpersonal synchrony and its subsequent effects on prosocial behavior among synchronized individuals. We hypothesized that areas of the brain associated with the processing of reward would be active when individuals experience synchrony during drumming, and that these reward signals would increase prosocial behavior toward this synchronous drum partner. 18 female non-musicians were scanned with functional magnetic resonance imaging while they drummed a rhythm, in alternating blocks, with two different experimenters: one drumming in-synchrony and the other out-of-synchrony relative to the participant. In the last scanning part, which served as the experimental manipulation for the following prosocial behavioral test, one of the experimenters drummed with one half of the participants in-synchrony and with the other out-of-synchrony. After scanning, this experimenter “accidentally” dropped eight pencils, and the number of pencils collected by the participants was used as a measure of prosocial commitment. Results revealed that participants who mastered the novel rhythm easily before scanning showed increased activity in the caudate during synchronous drumming. The same area also responded to monetary reward in a localizer task with the same participants. The activity in the caudate during experiencing synchronous drumming also predicted the number of pencils the participants later collected to help the synchronous experimenter of the manipulation run. In addition, participants collected more pencils to help the experimenter when she had drummed in-synchrony than out-of-synchrony during the manipulation run. By showing an overlap in activated areas during synchronized drumming and monetary reward, our findings suggest that interpersonal synchrony is related to the brain's reward system.

## Introduction

Humans are the only primates that spontaneously synchronize their voices and movements during music making and dancing [1], a behavior found across all cultures [2] and emerging early in human childhood [3]. One hypothesis claims that music and dance are culturally evolved tools for fostering group cohesion and commitment, thereby increasing prosocial in-group behavior and cooperation [4], [5], [6]. In fact, within a study comparing four different experimental groups of male adults, Anshel and Kipper (1988) showed that the members of the group, that had sung together, cooperated better in a prisoner's dilemma game and scored higher on a questionnaire on trust, than the members of the other groups that had either read a poem collectively, listened to music or watched a film together [7]. Likewise, Wiltermuth and Health (2009) demonstrated increased cooperation among students after joint singing, compared to no singing or forced “asynchronous” singing [8]. Similarly, Hove and Risen (2009) found that the degree of synchrony between participant and experimenter in a finger-tapping task correlates with subsequent affiliation ratings [9]. Finally, Kirschner and Tomasello (2010) showed that joint music making facilitates prosocial and cooperative behaviors already among four-year-old children [10].

Although interpersonal synchrony seems to be universally important, little is known about the neural basis of this phenomenon. Therefore, we ask here, how the prosocial effects of synchronized interpersonal activity are mediated by the human brain. Changing one's prosocial behavior after interpersonal synchrony requires a number of processes to take place. First, individuals must be capable of synchronizing their own actions to external stimuli. Furthermore, individuals must sense interpersonal synchrony and transform this synchrony into a reward-like signal that has the potential to shape future behavior. Finally, this signal must specifically affect prosocial tendencies towards the people one had acted in synchrony with. If any brain structure were common to these sub-processes, it would form an ideal candidate structure for linking synchronized activity to increased prosocial behavior. As we will see below, the caudate nucleus seems such a candidate associated with all these sub-processes and will therefore be at the core of our investigation.

First, studies investigating how we can synchronize actions to external stimuli found that, among others, the basal ganglia, which include the striatum (caudate and putamen), the pallidum and the substantia nigra, are important for our capacity to synchronize our actions to external stimuli [11], [12], [13], [14], [15]. Second, neuro-economists investigating which areas of the brain are associated with reward have shown that the striatum is activated by stimuli associated with both monetary [16], [17], [18], [19] and social reward (e.g. a gain in reputation) [17]. Third, studies using economic exchanges to manipulate trust and social risk taking [20], [21], [22], [23] have shown that the striatum and, in particular, the caudate also plays an important role in facilitating prosocial behavior [23]. Finally, an accumulating body of research suggests that the striatum is important in changing the likelihood of particular behaviors based on past reward [24], [25], [26] and reinforcement learning [27], [28]. Within the striatum, the caudate seems to be particularly sensitive to the contingency between an action or stimulus and its positive or negative consequences [26], thereby modulating future behavior based on reward history [29]. Moreover lesions to the caudate in animals were found to impair stimulus-response learning, i.e., prevent animals from appropriately changing their response to a stimulus after a past rewarding experience with that stimulus (see [28] for a review).

In summary, the caudate within the striatum appears to be one of the structures at the intersection of a number of important sub-processes that could link synchronized activity to processing of reward and future prosocial behavior. In this study we directly test this possibility specifically for the caudate. We investigated how experiencing synchrony during rhythmic musical actions is processed within the caudate, and how this modulates prosocial behavior at a later point in time. Based on the role of the caudate in synchrony and in reward processing, we hypothesized that synchrony during joint drumming triggers activity in the caudate. Based on the role of the caudate in reward-based stimulus-response learning and prosocial behavior, we hypothesized that the activity in the caudate during joint drumming then leads to a reinforcement of the association between the stimulus of the synchronized drum partner and future prosocial behavior.

## Methods

### Participants

18 healthy volunteers (all right-handed and female; mean age 23 years ranging 19–30 years) with normal or corrected to normal vision and without a history of neurological, major medical, or psychiatric disorders participated in the present study. Two participants of the initially 20 recruited participants were excluded from the study. These two participants reported at the end of the scanning that they suspected having not really drummed with the experimenters during scanning, undermining the social relevance of the manipulation. Only females were recruited in order to avoid possible gender confounds since the experimenter (I.K.) who performed the prosocial commitment test (see below) was female. None of the volunteers had any musical training or had ever played a musical instrument (except music classes at primary school). Participants gave their written informed consent and were paid for their participation. The experiment was approved by the Medical Ethical Commission of the University Medical Center Groningen, the Netherlands.

### Experimental procedure

Please see Supporting Information, Fig. S1A for the overall procedure. All participants performed: 1) a training session in which they were familiarized with a syncopated rhythm and their drumming task. Unknown to the participants, their performance was rated according to ‘ease of rhythm imitation’ during this session; 2) an fMRI scanning session with an fMRI localizer involving a monetary reward task to functionally define the specific regions of the caudate involved in reward processing; 3) an fMRI session consisting of 2 runs in which the participants believed that they were drumming with one of the experimenters in half of the blocks and with the other experimenter in the other half. To manipulate the experienced synchrony between the participants and their co-drummers, one co-drummer was drumming in- (*synch*) and the other out-of-synchrony (*asynch*); 4) a manipulation run in which participants believed that they were drumming only with I.K. (instead of alternating two experimenters) who either drummed in- or out-of-synchrony with the participant); 5) a prosocial commitment test outside of the scanner, immediately after the manipulation run, to assess the propensity to help I.K.

### Training

Before scanning, participants were familiarized with a syncopated rhythm consisting of 10 notes (Supporting Information, Fig. S1B) and learned to drum the rhythm by using a button box. The rhythm had to be played with the index fingers of both hands, starting with the left finger, followed after 600 ms by two right finger beats, one left and one right finger beat (each 300 ms long). After 900 ms, two left finger and two right finger beats (each 300 ms long) were followed by a last left finger beat (see Supporting Information, Fig. S1B for score and timing). The rhythm was introduced by a demonstration video presenting the rhythm two times successively performed by a male experienced drummer (not one of the two experimenters) with both index fingers on African bongos (Supporting Information, Video S1, 10 s long). In this video only the trunk, arms and hands of the drummer and a bongo on a table in front of him was visible. Each participant watched this video two times unless the participant asked for more repetitions. Participants were informed they could try to reproduce the rhythm using the left- and right-most button of an MRI compatible button box, while watching the demonstration video. Later they practiced the rhythm with the computer presentation program and the button box; the left- and right-most buttons were associated with two different prerecorded bongo sounds. The trial structure of the training trials was identical to that of the experiment (see Supporting Information, Fig. S1B).

During training, both experimenters rated the progress of the participants in acquiring the preset rhythm. Participants received a score ranging from 1 (needed assistance) to 5 (immediately able to reproduce the rhythm) based on the number of times the participant asked to watch the demonstration video, whether she asked for additional help from the experimenter and how early she managed to reproduce the rhythm (see Supporting Information, Table S1 for the detailed rating definition).

After the individual training, participants practiced with the experimenters. The participant and one experimenter sat next to each other. Participants used two buttons of a button box and the experimenter two buttons of a regular keyboard to control a computer. The computer presentation program associated different sounds to each of the found buttons, with the experimenter's buttons associated with lower tones than the participants' (Supporting Information, Listening Examples S1 and S2). Participants played the rhythm with one of the experimenters for three consecutive blocks (3×8 repetitions) and then repeated the same procedure with the other experimenter. To vary synchrony, for half of the participants, I.K. drummed in-synchrony with the participant whereas the other experimenter drummed out-of-synchrony with the participant (by delaying or anticipating the timing of corresponding button presses) on purpose (see, Supporting Information, Listening Example S2). For the other half of the participants, the roles of the experimenters were reversed: I.K. drummed out-of-synchrony and the other, in-synchrony with the participant. One of the experimenter was wearing a red t-shirt and the other a blue t-shirt. This was used to associate each experimenter with a color that could be used to let participants know with whom they were drumming during scanning. Performance while playing with the experimenters was not taken into account in the evaluation of the ‘ease of rhythm imitation’ rating in order to separate social factors studied later in the experiment from the assessment of people's individual aptitude.

### Scanning environment

During scanning, supine participants saw visual instructions projected via an LCD projector through a mirror positioned on the top of the head coil. All participants wore MRI-compatible headphones (MR confon GmbH, Magdeburg, Germany) without earplugs. All functional images were acquired on a Philips 3T scanner with the ‘soft-tone’ option turned on to reduce the gradient-noise and render the drums easier to hear. A conventional MRI-compatible response box (fORP, Current Designs, Inc., Philadelphia, USA) with 4 buttons was placed in front of the participants on a table so that they could use the box bimanually. The first and fourth (the left-most - red and the right-most - blue) buttons were used as bongos during the experiment (see Supporting Information, Fig. S1B); the second, third and fourth buttons (from left) were used during the localizer experiment. All Stimuli were programmed and presented using the software Presentation 12.0 (Neurobehavioral systems, Davis, CA, USA).

### Localizer Experiment

This gambling task (Fig. S1C) was adapted from the monetary reward task generously provided by Izuma and colleagues [17]. In each trial (3 s), the participants saw three cards with labels “A”, “B” and “C” side-by-side for a choice period of 2 s. Participants then had to choose one card by pressing the spatially corresponding button of their button box (using the right index, middle, or ring fingers). After the response, they saw the chosen card highlighted with a white border and the outcome (1 s). If the participant did not press any button within 2 s, the card they had chosen in the previous trial was automatically chosen, and its outcome was displayed. Blocks were constituted of 8 trials and lasted 24 s. Two types of blocks – reward and non-reward blocks - were distinguishable for the participant by the color of the labels on the cards. In monetary reward blocks, the outcome of choosing a particular card was randomly associated with 0, 0.30 or 0.60 EUR. In the no monetary reward (NMR) blocks, the outcome was always “XXX”, indicating no reward. Additionally, there were baseline blocks during which a red cross was presented instead of the cards. For half of the participants, the color of the letters signaling reward blocks was red and non-reward blocks, blue. For the other half, the color assignment was switched.

Unbeknown to the participants, the total amount one could earn in each monetary reward block was predetermined and defined as high or low: During a high monetary reward block (HMR), participants earned on average 3.3 EUR (range = 2.7–3.9 EUR), which was consistently higher than the expected value of eight reward trials (2.4 EUR). During a low monetary reward (LMR) block, they earned an average of 1.5 EUR (range = 0.9–2.1 EUR), which was consistently lower than the expected value. Two reward blocks were always separated by a NMR block or a rest block (a red cross). The localizer experiment comprised 4 runs (each run had five HMR, five LMR, five NMR and five baseline blocks) and lasted 8 min. Participants were told that at the end of the experiment, the computer would randomly choose one of the 4 runs, and they would be paid the amount they had earned on that run. This ensured that the game had significant financial consequences for the participants.

### Drumming Experiment

The task of the participants was to play the rhythm they practiced in the training session as correctly as possible. They were explained that the two experimenters, one wearing a red, the other a blue T-shirt, would both sit in the control room. As in the last part of the training, one of the experimenters would drum with the participant for one block of trials, then the other experimenter would do the same, and so on. A colored square on the T-shirt of the drummer in the demonstration video indicated with which of the experimenter the participant would be drumming during that block. However, in order to standardize our experimental conditions, the co-drumming in each trial was computer simulated during the experiment. Importantly, participants were not instructed or encouraged either to drum in synchrony with the experimenters or to attend to the drums of the co-drummer. Their task was simply to play their own rhythm as accurately as possible.

During scanning, each block started with a demonstration video (10 s, Supporting Information, Video S1, described in detail in the *Training* section) followed by 8 trials. 300 ms after the end of the video, the numbers “3”, “2” and “1” appeared on the screen, indicating the pulse of the rhythm. Each number was presented for 300 ms and successive numbers were separated by 300 ms of black screen. Participants were instructed to start playing their drums whenever they saw the number “1” on the screen (appearing 1500 ms after the end of the video). In order to help participants to keep the beat across repetitions of the rhythm, they saw the number “3” on the screen when they had to play the last note of the rhythm, followed by 300 ms of black screen, 300 ms of “4”, and 300 ms of black screen to ensure a total of 900 ms of silence between repetitions of the rhythm. After that, the new trial started with the presentation of number “1” (300 ms) to cue a new instance of the rhythm (see Supporting Information, Fig. S1B for the time course of a trial). These numbers served as indications for beats of a 4/4 bar at the tempo 100 beats per minute. Participants learned to use these visual instructions in the training session and were told that the co-drumming experimenter would also use these visual instructions. Blocks within the experiment comprised two different conditions:

1. *Synchronous Drumming (synch) Block*: Participants played 8 trials of the rhythm and were lead to believe that they did so together with the experimenter they had experienced as synchronous during the training before scanning. In reality, a program was used to simulate the synchronous experimenter by presenting the correct notes after a randomized 15–75 ms interval following the button press of the participant (Supporting Information, Listening Example S1). This was done to simulate a synchronous drummer adapting his/her beat to that of the participant within a tight but varying time window resulting in a natural sounding synchronous drumming. Using only positive time delays relative to the participant ensured that the participant could not entrain to the experimenter.
2. *Out of Synchrony Drumming (asynch) Block*: Participants played 8 trials of the rhythm and were lead to believe that they drummed together with the co-drumming experimenter who had not been in synchrony with the participants during the training. In reality, the program presented different prerecorded rhythms randomly in this block (Supporting Information, Listening Example S2). These prerecorded rhythms were composed by randomly shifting the timing of the original notes of the sequence (−400 to +300 ms). In piloting the experiment, this jitter was perceived as corresponding to a drummer unable to keep the beat while preserving the overall structure of the rhythm.

All blocks were separated by 14±2 s random pauses (baseline) with a red cross presented in the center of the screen. In total, the experiment consisted of three runs. The first two runs lasted 16 minutes and each contained 4 *synch* and 4 *asynch* blocks in pseudo-random order counterbalanced between runs and participants. The last run was designed as a manipulation run: only I.K. drummed with the participants for five blocks. She was the in-synch drummer for half and the out-of-synch drummer for the other half of the participants. Importantly, because I.K. needed to conduct the prosocial commitment test after scanning, she had not to know whether she had (supposedly) been in- or out-of-synch with the participant in this last scanning run. The other experimenter therefore randomly picked a program that simulated an in- or out-of-synch drummer (as described above) for this last run. Thus, during initial training and during the prosocial commitment test, I.K. was blind to the way she supposedly drummed during this manipulation run. Naturally, this randomization led to 4 different possible histories: 2 role-switch configurations in which I.K. was in-synch during the training and the first 2 runs of the fMRI experiment but out-of-synch during the manipulation run or vice versa and two no-role switch configurations in which she was in-synch or out-of-synch throughout the experiment. We had 3 participants in each of the role-switched groups (initially, we had two more participants in the role switch groups, however two of those participants were excluded, see description *Participants*) and we had 6 participants in each of the no-role switch groups.

### Prosocial Commitment Test

This test was performed to measure the prosocial commitment of the participants towards I.K. depending on whether she had drummed - according to the experimental condition - in- or out-of-synchrony with the participants in the manipulation run. The participants were not aware that this was a test and I.K. did not know if she had been in- or out-of-synch during the manipulation run. Immediately after the end of scanning, participants were asked to fill out a questionnaire and were guided by I.K. to the waiting room. This room was empty except for a table along one side of the room. She explained that she would leave to bring a chair and pencils so that the participant could sit down and fill out the questionnaire. After a minute, she came back, holding a chair with both hands and a plastic cup containing 8 pencils in one hand. The moment she entered the room, she pretended to accidentally drop the plastic cup, such that all eight pencils fell on the floor and spread around the room. Her hands were busy carrying the chair when she dropped the pencils and it took her about 10 s to place the chair in front of the table. Given that the distance between the various pencils and the participant varied considerably, in this 10 s time window participants could decide how many pencils they would pick up to help the experimenter: none, only those within close reach, or even those requiring the participant to walk around to pick them up - leading to a relatively continuous dependent variable. As a measure of the participant's prosocial commitment towards the experimenter, we therefore counted the number of pencils that the participant picked up. Please see [30], [31] for similar prosocial tests and validation of its use for measuring helping behavior [30]. After that, the participant filled out a questionnaire about the experiment. With this questionnaire we surveyed how difficult and enjoyable the participants found the experiment during scanning (e.g., “ How much did you like drumming with the person who was wearing a red t-shirt?” on a 1 = very little to 5 = very much scale. All questions can be found in the Supporting Information, Table S2).

### Behavioral Data Analysis

#### Drumming Performance during Scanning

We evaluated our non-musician participants' performance during scanning by analyzing the onsets of the participants recorded button presses.

### fMRI Data Analysis

#### Data acquisition

Imaging was performed with a Philips Intera 3T Quaser with a synergy SENSE eight channel sense head coil and maximum gradient strength of 30 mT/m with a soft tone sequence. Head movements never exceeded 3 mm in a run. We used a standard single shot EPI with TE = 27 ms, TA = 1.45 s, TR = 1.5 s. For each volume, 30 AC-PC aligned axial slices of 4 mm thickness, without slice gap and a 3.5×3.5 mm in plane resolution were acquired to cover the entire brain using an interleaved slice acquisition. A T1 weighted structural scan was acquired with TR = 9 ms, TE = 3.53 ms, flip angle = 8 deg.

#### Data preprocessing

We used SPM5 (www.fil.ion.ucl.ac.uk/spm) implemented in MATLAB 6.5 (Mathworks Inc., Sherborn, MA, USA) for fMRI data analysis. All EPI volumes were aligned to the first volume acquired for each participant and a mean EPI image was generated after realignment. Spatial normalization was performed by co-registering the structural volume to the mean EPI, segmenting the co-registered structural image, determining the normalization parameters required to warp the gray matter segment onto the gray matter MNI template, and applying these parameters to all EPI and structural volumes. Normalized images were written with an isotropic resolution of 2 mm for EPI and 1 mm for structural images. The normalized EPI images were smoothed with an 8 mm FWHM isotropic Gaussian kernel. The normalized structural images (T1) were then averaged across participants for visualization of results. The preprocessing of the experiment and the localizer task was done with same procedure and the same normalization parameters.

#### General data analyses

Functional data were analyzed using a general linear model (GLM, see Supporting Information, Table S3 for abbreviations) separately for each participant and voxel using SPM5. We modeled the data in a block design fashion. Each block consisted of 8 trials; in total each participant performed 8 blocks of 8 trials per condition (64 trials total). Although there were mistakes in some trials in the blocks, the number of trials with mistakes for all 8 blocks combined was very low (mean = 2.11/64 trials in the *synch* condition; mean = 3.06/64 in the *asynch* condition; see Results, Behavioral Results). Given that on average 97- 95% of the trials were therefore without mistakes, we decided not to exclude any trials or blocks from the analysis. The localizer task was modeled in a separate design matrix in a block design fashion.

#### Single participant analyses

For the drumming experiment, the GLM was performed using separate predictors for the conditions *synch*, *asynch* and the video (demonstration video, which was shown in the beginning of each block). Likewise, for the localizer task, the GLM was performed for the HMR, LMR and NMR predictors in a separate design matrix. Each predictor was a boxcar function that reflected the length of the block. The boxcar functions were convolved with the hemodynamic response function, and fitted separately for each run to the data. In addition, the head motion and rotation along the three axes were entered as 6 covariates of no interest in the design matrix to single out motion artifacts although motion never exceeded 3 mm within a run.

#### Population analyses

At the second level analysis, the contrast images from the first level analyses of the single participants were entered into random-effects models (RFX) to make inferences at a population level. Group analyses were thresholded at the voxel-level at p<0.005 (uncorrected) with a minimum cluster size of 10 voxels. To control the overall rate of false positives, only results also surviving a False Discovery Rate correction (FDR) of p<0.05 (and a minimum cluster size of 10 voxels) are reported. This double procedure was used instead of only using an FDR correction, or only using an uncorrected threshold for the following reasons. Actual t-thresholds vary considerably depending on the size of the search space when employing only FDR correction. Thus, comparing activations in different contrasts or comparing whole brain and region of interest (ROI) analyses would be difficult due to these different t-thresholds. Only using an uncorrected threshold brings the risk of excessive false positives in larger search volumes because of the multiple comparison problems. Calculating the critical t-value for both methods and using the more stringent of the two however ensures that all results are protected against excessive false positive rate while at the same time imposing a similar minimal requirement of p<0.005 even in small ROIs.

For the monetary reward localizer, whole brain analyses were performed. Most of the analyses regarding the drumming experiment were conducted in the ROI only to provide maximum power to test our hypothesis.

In order to localize reward sensitive regions of the caudate, HMR-NMR contrast images of the monetary reward task of the single participants were entered into one-sample t-tests to instantiate a random-effects group analyses. On the basis of the literature emphasizing the role of the caudate in interpersonal synchrony, reward and prosocial behavior, we aimed to specifically test the involvement of this ROI. We therefore multiplied the thresholded and binarised group t-map of the localizer described above with a binary volume containing ones in the caudate and zeros elsewhere (obtained from the WFU Pick Atlas Tool, http://www.fmri.wfubmc.edu/download.htm) using the ImCalc function in SPM. Later, the resulting overlap image was used as a caudate-reward mask in order to perform small volume corrections for the results of the drumming experiment to explore the involvement of reward related caudate in our task.

For the drumming experiment, we performed the random-effects group analyses using t-tests for the contrasts *synch* - baseline, *asynch* - baseline, *synch - asynch* and *asynch - synch*.

Two sets of multiple regressions on the second level were performed: Due to the substantial differences detected in ease of rhythm imitation across participants in the training period, we used ‘ease of rhythm imitation’ as a covariate in order to analyze the link between participant's ease of rhythm imitation and their brain activity during synch vs. asynch drumming. Similarly, we employed a regression analysis with number of pencils picked up in the prosocial test as covariate, in order to explore the brain regions showing correlation between synchronous or asynchronous drumming in the first two runs of the drumming experiment, and the number of pencils participants collected after scanning to help the in-synch or out-of-synch experimenter of the manipulation run, respectively.

## Results

### Behavioral Results

#### 1) Drumming Performance during Scanning. Mistakes

Trials of drumming (in total 128 trials: 64 trials in each condition) were inspected for three types of mistakes done by the participant: missing a note, stopping to play after several notes or skipping an entire trial or playing the rhythm wrongly, mainly by playing the wrong beats (see Supporting Information, Table S4). A three (mistake type)×two (condition: experienced asynchronous or synchronous drumming) repeated measures ANOVA revealed neither a significant main effect of mistake type (F_(2,34)_ = 3.56, p = 0.07) nor of drumming condition (F_(1,17)_ = 2.94, p = 0.11), nor an interaction between the type of mistake and drumming condition (F_(2,34)_ = 2.05, p = 0.15, for all p values the Greenhouse-Geisser sphericity correction was applied) on the number of trials with mistakes.

#### Individual Beat

Comparing the timing of each button press of the participant with the timing of the button presses that were requested by the given rhythm, indicated that all participants demonstrated negative asynchronies (i.e., their button presses were before the “requested” time) in each trial (first note is taken as start of the rhythm and has a 0 ms asynchrony and is not taken into account). The mean and standard deviation of asynchronies relative to the demonstrated rhythm can be found in the Supporting Information, Table S5. By averaging the mean accuracies over all 9 notes per trial we found marginal significant differences (t_(17)_ = −2.0, p = 0.059) between the *synch* condition (mean ± SD: −48.6±16.9 ms) and in the *asynch* condition (mean ± SD: −43.7±15.2 ms). Analyzing the variability of the beats of the participants we also averaged the standard deviations of the mean asynchronies of each participant button presses over all 9 notes per trial. Participants were more variable (t_(17)_ = −5.5, p<0.001) in drumming in the *asynch* condition (mean of SD ± its SD: 45.2±9.0 ms) than in *synch* condition (mean of SD ± its SD: 30.2±8.7 ms). The more detailed analysis, a 9×2 repeated measures ANOVA (mean asynchrony of all 9 individual notes×condition), tested if there were timing differences in the beats played by participants within the different drumming conditions (*synch* or *asynch*). There was the already identified marginal main effect of condition (F_(1,17)_ = 4.1, p = 0.059). Furthermore, we found a main effect of the note (F_(8,136)_ = 230.8, p<0.001) and significant interaction between the note and condition (F_(8,136)_ = 13.7, p<0.001, for all p values the Greenhouse-Geisser sphericity correction was applied). The same 9×2 repeated measures ANOVA was performed on the standard deviations (standard deviation of mean asynchronies of each individual note×condition) in order to test whether there were variability differences in the beats played by participants between the different drumming conditions. We found a main effect of condition, note and an interaction (condition: F_(1,17)_ = 30.8, p<0.001; note: F_(8,136)_ = 46.2, p<0.001; interaction: F_(8,136)_ = 4.6, p<0.01, for all p values the Greenhouse-Geisser sphericity correction was applied). Please see Supporting Information, Table S5 for the means and the standard deviation of the asynchronies of the participant's button presses relative the demonstrated rhythm.

#### 2) Ease of Rhythm Imitation

Participants' ease of rhythm imitation was evaluated during training according pre-defined criterion on 5 point scale (1 = needed assistance, 5 = immediately able to reproduce the rhythm; see Supporting Information, Table S1). The mean ease of rhythm imitation rating was 3.1 (SD = 0.98) on that 5 point scale with substantial differences across participants. The scores of the ease of rhythm imitation of single participants correlated with their number of mistakes during drumming during scanning (r = −.45, p<0.05, one tailed), their perceived difficulty of the rhythm (r = −.45, p<0.05, one tailed), and their self-judged concentration needed to play the rhythm (r = −.52, p<0.05, one tailed). Thus, participants who acquired the rhythm easier and faster (higher numbers) made less mistakes during scanning, experienced the rhythm as being less difficult and they reported to have needed less concentration to drum than those participants who had more difficulties to learn to drum the rhythm.

#### 3) Prosocial Commitment Test

**Figure 1:**
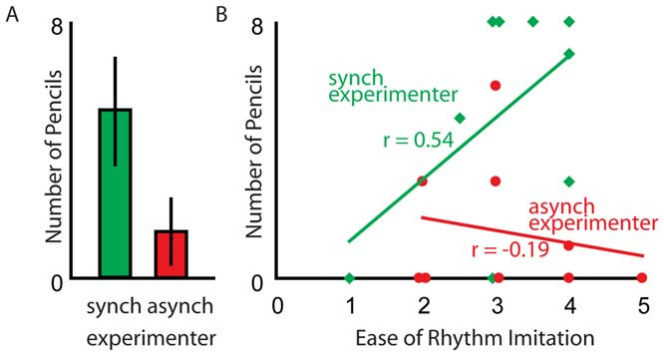
Prosocial commitment test results (A) and the ease of rhythm imitation (B). (A) Mean (and standard deviation) of the number of pencils picked up in order to help I.K. in the prosocial commitment test after experiencing her as a synchronous (green) or asynchronous drummer (red) in the manipulation run. (B) Correlation between the number of pencils and the ease of rhythm imitation as a function of synchrony (green: I.K. who played in-synch; red: she played out-of-synch during the manipulation run). The lines represent the linear best fit and r refers to the correlation coefficient.

Participants collected more pencils when I.K. ‘accidentally’ dropped 8 pencils in front of the participants when she had been a synchronous drum partner (mean = 5.22, SD = 3.42 pencils) compared with when she had been an asynchronous drum partner (mean = 1.44, SD = 2.13 pencils) in the manipulation run (see Fig. 1A). This difference in helping effort was highly significant between conditions (t (16) = 2.8, p<0.05), demonstrating more prosocial commitment towards the experimenter if she had drummed synchronously in the manipulation run, right before dropping the pencils.

To investigate if this effect was primarily due to the role of the experimenter in the manipulation run, we analyzed the number of pencils picked up using a 2×2 ANOVA (*sync* or *async* during the first two runs of the drumming experiment sync vs. async during the (third) manipulation run). This revealed a significant main effect of role played in the manipulation run (F_(1, 17)_ = 6.4, p<0.05), but not for the role played in the first two runs of the drumming experiment (F<1), and the interaction was not significant (F<1). To further investigate the role of the experimenter during the first two runs and the manipulation run, we additionally calculated several correlations (see Supporting Information S1 and Table S2, for more details). The post-scanning questionnaires revealed that participants had processed and attended the color of the t-shirts of the experimenters and matched the color of the t-shirts with the color of the square in the demonstration video (indicating the drum partner) before each drumming block during scanning. This was evident from participants post-scanning questionnaires (see Supporting Information Table S2 for details). Participants' report of how much fun it had been to play with a particular colored experimenter depended both on the role played by that experimenter during the first two scanning runs (Pearson's r = 0.64, p<0.01) and during the third, manipulation, run (Pearson's r = 0.51, p<0.05). The same was true for reports of how much they liked that particular colored experimenter (Pearson's r = 0.80, p = 0.001 and Pearson's r = 0.66, p<0.001, respectively). These positive correlations further indicate that participants not only helped the synchronous experimenter more but also experienced more fun and liked drumming more with the in-synch experimenter. This provides further evidence that processing of reward might be triggered by synchronous activity.

#### 4) The Interaction between the Ease of Rhythm Imitation in the Training and the Prosocial Commitment

Because of the variability in ease of rhythm imitation across our participants, we explored if there is a relation to prosocial commitment after participants experienced more or less synchronous drumming. We found a marginally significant positive correlation between participants' ease of rhythm imitation and the number of pencils collected to help the synchronous experimenter of the manipulation run (Pearson's r = 0.54, p = 0.065, one-tailed; Fig. 1B, green). On the other hand, we did not find such a correlation between the ease of rhythm imitation and the helping behavior towards the asynchronous experimenter of the manipulation run (Pearson's r = −0.19, p = 0.31; Fig. 1B, red). Note that every participant was only tested once in the pencil test because two different experimenters dropping pencils would have been conspicuous. Accordingly only half of our participants were tested with a synchronous experimenter and the other half with the one who was not in synchrony, resulting in reduced power by leaving only 9 participants in each subset. This result should thus be replicated in larger groups in future studies.

### Imaging Results

#### 1) Localizer Task

**Figure 2:**
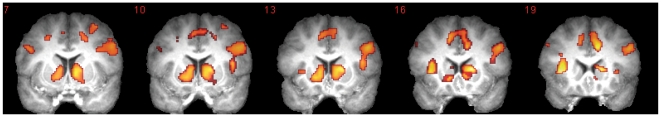
Areas of the brain associated with processing of monetary reward (contrast High Monetary Reward - No Monetary Reward; p<0.005 uncorrected, all voxels also survive p<0.05 FDR correction). Clusters are superimposed on to the average T1 image derived from all participants.

We mapped the brain areas involved in monetary reward processing by contrasting the HMR (High Monetary Reward) condition with the NMR (No Monetary Reward) condition. As expected, we found significantly more activation in the bilateral caudate when participants received monetary rewards. Additional activations were found in the right pallidum, right thalamus, bilateral insula, right supplementary motor area (SMA), right middle cingulate, right middle frontal gyrus, the bilateral precentral gyrus, bilateral inferior frontal gyrus, bilateral inferior and superior parietal lobule, the left lingual gyrus, the right middle occipital gyrus, left lingual gyrus and the cerebellar vermis. (t(17)>2.9, p<0.005 uncorrected, all clusters also survive p<0.05 FDR correction (see Figure 2 and Table 1). Our results were in accordance with the previous findings on monetary reward processing [16], [17], [18], [19].

**Table 1:** Results of the contrast between the High Monetary Reward and No Monetary Reward (HMR>NMR).

Size (vox)	Hem	Area	x	y	z	t
2331	R	Caudate	14	18	4	6,14
	R	Pallidum	12	4	−4	5,54
	R	Thalamus	10	−8	6	4.68
	L	Caudate	−8	10	−2	4.65
	R	C.Vermis	6	−42	−20	4.19
1048	R	hIP1	32	−52	30	4,64
	R	Ang Gyrs/SPL	32	−66	44	4,53
	R	supMGyrs	48	−40	32	4,18
	R	MOG	34	−66	32	3,88
958	L	IPL	−30	−62	38	4,49
	L	SPL	−18	−64	40	4,03
	L	Precuneus	−12	−66	38	3,59
	R	Precuneus	8	−62	48	3,53
	L	supMGyrs	−44	−44	32	3,17
845	R	preCG (Area 6)	30	−4	44	5,04
	R	MFG	32	8	56	3,58
672	R	midCingulate Crtx	12	20	32	5,28
	R	SMA	4	22	46	4,13
591	R	IFG	46	10	32	3,64
507	L	preCG	−46	−4	30	5,02
337	L	Insula	−34	18	2	5,87
	L	IFG	−40	24	26	3,84
99	R	Insula	34	22	0	4,01
60	L	Lingual Gyrus	−20	−74	−8	3,88

Results of HMR>NoMR at p<0.005 uncorrected (all voxels also survive false discovery rate correction at p<0.05). Only clusters of 10 voxels or more are reported. For each cluster, its size in voxels and hemisphere are indicated first. For each of the subpeaks of the cluster, the cytoarchitectonic areas (based on the anatomy toolbox [38] for SPM) followed by their MNI coordinates and t-value are reported. See Table S3 for abbreviations.

#### 2) Drumming Experiment. Synchronous and Asynchronous Drumming

Before examining our hypothesis in the ROI (see Methods), we first performed a whole brain analysis to map brain regions recruited during the various drumming conditions. We indentified the following brain areas to be activated by drumming with a co-drummer who was in synchrony with the participant (*synch*>baseline: t (17)>2.9, p<0.005 uncorrected; all clusters also survive p<0.05 FDR correction): the right superior temporal gyrus (auditory cortex), the left middle temporal gyrus, bilateral postcentral gyrus, right inferior parietal lobule, right inferior frontal gyrus (Brodmann Area, BA44), right SMA, the bilateral pallidum, right caudate, bilateral thalamus, left putamen and right cerebellar vermis (see Table 2).

**Table 2:** Results of the *sync*h-baseline contrast.

Size (vox)	Hem	Area	x	y	z	t
21508	L	postCG	−52	−16	40	9,08
	R	IFG (BA 44)	52	6	14	8,26
	L	MTG	−54	−38	8	8,10
	R	C.Vermis (III)	2	−36	−16	7,97
	R	SMA	4	−2	52	7,87
	R	postCGyrs (Area 4p)	38	−26	52	7,86
	R	STG/TE 1.1	50	−14	−4	7,49
	L	postCGyrs (Area 2)	−42	−32	42	7,45
	R	IPL	44	−46	48	7,44
	R	supMGyrs	54	−34	42	7,44
3101	L	Pallidum	−20	−4	2	6,24
	R	Pallidum	20	−6	−4	5,55
	R	Caudate	14	6	8	4.65
	R	Thalamus	12	−12	4	5,29
	L	Thalamus	−12	−14	4	5,28
	L	Putamen	−18	4	8	4,81

Results of *synch*-baseline at p<0.005 uncorrected (all voxels also survive false discovery rate correction at p<0.05). Only clusters of 10 voxels or more are reported. For each cluster, its size in voxels and hemisphere are indicated first. For each of the subpeaks of the cluster, the cytoarchitectonic areas (based on the anatomy toolbox [38] for SPM) followed by their MNI coordinates and t-value are reported. See Table S3 for abbreviations.

The areas that were activated while drumming with a co-drummer who was not in synchrony with the participant (*asynch*>baseline: t (17)>2.9, p<0.005 uncorrected; all clusters also survive p<0.05 FDR correction) included the right auditory cortex, left middle and superior temporal gyrus, right post central gyrus, bilateral inferior parietal lobule, left superior parietal lobe, right inferior frontal gyrus (BA44), right pallidum, right putamen, left thalamus, and right cerebellar vermis (Table 3).

**Table 3:** Results of the *asynch*-baseline contrast.

Size (vox)	Hem	Area	x	y	z	t
16489	L	MTG	−52	−38	8	9,38
	R	STG/TE 1.0	52	−10	−6	9,23
	R	IFG (BA 44)	52	6	14	8,61
	R	suprMarg Gyrs	52	−36	44	8,38
	R	postCGyrs (Area 3b)	44	−18	46	7,94
	L	IPL	−40	−30	40	7,62
	R	postCGyrs (Area 4p)	38	−26	52	7,36
	L	STG	−62	−22	12	7,31
	R	C.Vermis (I/II)	2	−36	−16	7,97
	L	Thalamus	−14	−14	−2	7,33
	R	Putamen	24	16	0	6,00
	R	Pallidum	22	4	2	5,98
36	L	SPL	−30	−60	42	3,82

Results of *asynch*-baseline at p<0.005 uncorrected (all voxels also survive false discovery rate correction at p<0.05). Only clusters of 10 voxels or more are reported. For each cluster, its size in voxels and hemisphere are indicated first. For each of the subpeaks of the cluster, the cytoarchitectonic areas (based on the anatomy toolbox [38] for SPM) followed by their MNI coordinates and t-value are reported. See Table S3 for abbreviations.

Using corrections for multiple comparisons (FDR, p<0.05) within the entire brain, as above, neither the contrast *synch*>*asynch* nor *asynch*>*synch* revealed significant differences.

### Ease of Rhythm Imitation during Training and Synchronous Drumming

**Figure 3:**
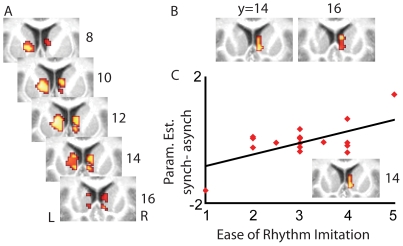
Correlations between the activity in the caudate and the ease of rhythm imitation. (A) Bilateral caudate activity correlated with the ease of rhythm imitation during synchronous drumming (*synch*-baseline, p<0.005 uncorrected, all voxels also survive p<0.05 FDR correction) (B) Right caudate activity correlated with the ease of rhythm imitation for the comparison between synchronous and asynchronous drumming (*synch - asynch*, p<0.005, uncorrected; all voxels also survive p<0.05 FDR correction) (C) Illustration of the correlation identified in (B) by plotting parameter estimates within the cluster against the ease of rhythm imitation rating. The line represents the linear best fit. Clusters in the caudate are superimposed on coronal views of the average T1 image derived from all participants.

Participants differed substantially in how easily they learned to reproduce the rhythm during training. We reasoned that the ease with which participants drummed might influence how open they are to what the other drummer plays. Accordingly, we assessed whether individual differences in the training covaried with brain activity in our ROI for reward, the caudate, during synchronous drumming. Figure 3A shows that those participants who had more ease at reproducing/imitating the rhythm before scanning activated the bilateral caudate more during synchronous drumming (second level regression analysis between brain activity during *synch* and ease of imitation; t (16)>2.9, p<0.005 uncorrected and p<0.05 FDR corrected within the ROI, Table 4). Furthermore, the ease of rhythm imitation covaried with the activity in the right caudate for the synchronous drumming more than for the asynchronous drumming (second level regression analysis between the contrast *synch-asynch* and ease of rhythm imitation; t(16)>2.9, p<0.005 uncorrected and p<0.05 FDR corrected within the ROI, Table 5 and Fig. 3B). To illustrate this relation more extensively, we have extracted the parameter estimates of that activation cluster for each of the 18 participants and have plotted this together with the scores for ease of rhythm imitation (Fig. 3C).

**Table 4:** Caudate correlating with the ease of rhythm imitation for the *synch* – baseline contrast.

Size (vox)	Hem	Area	x	y	z	t
56	L	Caudate	−6	10	−2	3.7
14	R	Caudate	10	10	6	3.29

Correlation of brain activity in the caudate (ROI) with the ease of rhythm imitation for the *synch*>baseline contrast at p<0.005 uncorrected (all voxels also survive false discovery rate correction at p<0.05). Only clusters of 10 voxels or more are reported. For each cluster, its size in voxels and hemisphere are indicated first. For each peaks of the cluster, the MNI coordinates and t-value are reported. See Table S3 for abbreviations.

**Table 5:** Caudate correlating with the ease of rhythm imitation for the *synch*>*asynch* contrast.

Size (vox)	Hem	Area	x	y	z	t
33	R	Caudate	6	14	4	3.8

Correlation of brain activity in the caudate (ROI) with the ease of rhythm imitation for the *synch*>*asynch* contrast at p<0.005 uncorrected (all voxels also survive false discovery rate correction at p<0.05). Only clusters of 10 voxels or more are reported. For each cluster, its size in voxels and hemisphere are indicated first. For each peaks of the cluster, the MNI coordinates and t-value are reported. See Table S3 for abbreviations.

In order to test whether the inverse contrast would reveal significant results, we calculated a second level regression analysis between brain activity during a*synch-synch* and ease of rhythm imitation within a whole brain analysis (since we had no hypothesis for this). We found only the right amygdala (MNI coordinates of the peak: x = 38; y = −6, z = 26; T = 4.39, cluster size, 14 voxel) in this analysis (t(16)>2.9, p<0.005 uncorrected, see Supporting Information S2 for a discussion of this finding).

One might propose two alternative accounts of the correlation between caudate activity and ease of learning. Based on the role played by the caudate in synchronization, one might argue that it is because some participants have more activation in the caudate that they were faster at acquiring the rhythm initially. Alternatively, one might argue that people that learned the rhythm more easily struggle less to play the rhythm during the experiment, and might thus be more open to enjoying the rewards of synchronous playing with a co-drummer. The former, but not the latter explanation would predict that caudate activity correlates with the precision of the participants' drumming during scanning. To disentangle these accounts, we used circular statistics using the Rayleigh test. Circular statistics are a common procedure in the tapping literature for calculating the degree of synchronization of the individual's responses to an external rhythm in order to detect the variance of asynchronies from different trials [32] (the detailed explanation of the calculations can be found in [3]). We used the mean resultant length (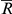) of each participant from this analysis, which assesses the mean variance of asynchronies in keeping the beat, as a regressor in order to test whether the caudate was more active in the better beat keepers. The analysis did not reveal significant correlations (positive or negative) between brain activity in the caudate during *synch*, *asynch* or *synch*-*asynch* and beat-keeping (p<0.005) and therefore speaks against the interpretation that more caudate activation was the cause for swifter learning rather than a result of increased sensitivity to synchronicity in less effortful playing. This negative finding does not seem to be due to a lack of power in the analysis: at the same threshold, brain activity in the *synch*-*asynch* contrast did correlate significantly with beat-keeping in a number of areas outside the ROI, including the inferior frontal and precentral gyrus.

#### 3) Prosocial Commitment

**Figure 4:**
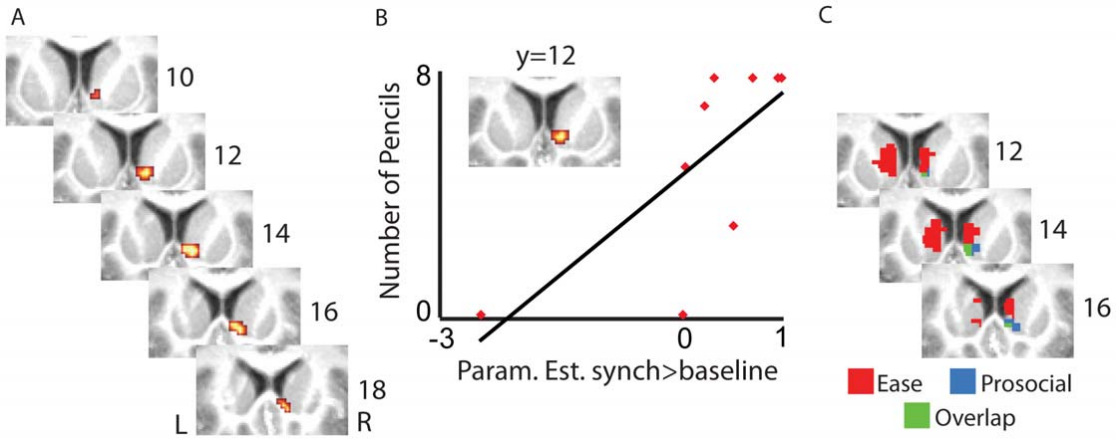
Correlations between the activity in the caudate and the prosocial commitment test. (A) Right caudate activity during synchronous drumming correlated with the number of pencils collected for the synchronous drummer (*synch*-baseline, p<0.005, uncorrected; all voxels also survive p<0.05 FDR correction) (B) Illustration of the correlation identified in (A) by plotting average parameter estimates within the cluster against the number of pencils. The line represents the linear best fit. (C) The overlap (green) of the correlation between brain activity during *synch* drumming and ease of rhythm imitation (red) and brain activity during *synch* drumming and number of pencils picked up (blue) (p<0.005, uncorrected; all voxels also survive p<0.05 FDR correction). Clusters in the caudate are superimposed on coronal views of the average T1 image derived from all participants.

As previously reported (see Behavioral Results), we found an influence of experiencing synchronous drumming during the manipulation run on the prosocial commitment towards the (synchronous) drum partner (see Behavioral Results, Prosocial Commitment Test). To examine the role played by the caudate in this prosocial behavior, we assessed whether brain activity in the caudate during the first two runs of drumming in the scanner could predict the number of pencils participants collected after scanning. for the synchronous or asynchronous experimenter. Figure 4A shows that activation in the right caudate (see Table 6 for coordinates) while experiencing synchronous drumming during scanning predicted the number of pencils collected after the scanning to help the synchronous experimenter of the manipulation run (multiple regression analysis between *synch-baseline* and number of pencils, second level; t(14) = 3.0, p<0.005, Table 6). The results survived the FDR correction (p<0.05) within the ROI. To illustrate this result, we extracted the parameter estimates from that analysis and plotted them against the number of pencils picked up (Fig. 4B). Statistical inferences based on the extracted data would be biased by “double dipping” [33] given that these parameter estimates were derived from the activation cluster that was determined using a statistical parametric map testing for the same contrast. Because the illustration suggests the presence of an outlier, it should be noted that excluding the outlier leads to Pearson's r = 0.63 and this would be a significant result (p<0.05, one tailed, 8 participants) if the data had not been selected to be significant for all 9 participants. No significant correlation was found between brain activity in the caudate during experiencing asynchronous drumming and number of pencils picked up for the asynchronous experimenter after scanning.

**Table 6:** Caudate correlating with the number of pencils for the *synch*–baseline contrast.

Size (vox)	Hem	Area	x	y	z	t
29	R	Caudate	10	12	6	3.5

Correlation of brain activity in the caudate (ROI) with the number of pencils for the *synch*>baseline contrast at p<0.005 uncorrected (all voxels also survive false discovery rate correction at p<0.05). Only clusters of 10 voxels or more are reported. For each cluster, its size in voxels and hemisphere are indicated first. For each peaks of the cluster, the MNI coordinates and t-value are reported. See Table S3 for abbreviations.

Finally, we found that the activity in the right caudate which correlated with the ease of rhythm imitation before the scanning was overlapping with the activity that correlated with the prosocial commitment after scanning (Fig. 4C). Thus, the less effort it cost a participant to produce the rhythm initially, the more activation was later found in the right caudate for synchronous drumming, and the more pencils the participant collected after scanning to help the experimenter who drummed synchronously.

## Discussion

The present study is the first to investigate the neural link between synchrony in joint drumming and prosocial behavior. Based on previous studies showing that synchronizing, reward and prosocial behavior all involve the caudate in the human brain, we (1) functionally localized that brain area with a reward task, (2) measured brain activity while manipulating the degree of synchronicity between a participant and an experimenter in a drumming task performed in the scanner, and (3) examined the impact of synchronous or asynchronous drumming on the participant's propensity to help the drum partner. Our results suggest that those participants, who mastered the rhythm more easily prior to scanning, showed increased activity in our region of interest (the bilateral caudate) when the drum partner drummed in synchrony with them. Moreover, the amount of activity in the right caudate during synchronous drumming predicted the level of prosocial commitment, measured by the number of pencils picked up by participants in a pencil-dropping test after scanning. In addition, participants who drummed with a ‘synchronous’ drum partner in the last part of the experiment showed more prosocial commitment towards this drum partner than those who drummed with an ‘asynchronous’ drum partner. These effects were stronger in participants that acquired the rhythm more easily. In the following we will discuss our results suggesting that synchronous drumming is socially rewarding and facilitates prosocial behavior between the synchronized individuals.

First, the analysis of the behavioral data and inspection of the number of trials with mistakes during drumming showed that participants were able to drum the rhythm in both conditions (*synch* and *asynch*) although they were slightly more variable in the asynchronous condition. This increased variability is not surprising given that in the asynchronous drumming condition, the beats of the experimenter were out of time in relation to the given rhythm and the participant's drumming therefore functioned as a distracter (see [34]). All participants tapped the individual beats before the expected time, which is consistent with the negative asynchronies found in many previous tapping studies, and which is even more pronounced in non-musicians (see [13], [35]).

Second, the analysis of ease of rhythm imitation during training prior to scanning showed that the participants differed in time and support needed to imitate or reproduce the novel rhythm. Although, after training all participants were able to drum the rhythm, those that had needed more assistance during the initial training continued to make more mistakes during scanning, suggesting a certain continuity between the ease of acquisition and the ease of drumming during scanning. Accordingly, we hypothesized that those participants who required more assistance initially would need to remain more focused on their own drumming during scanning. Because Chapin and colleagues (2010) measured stronger activity in the caudate to auditory presented syncopated rhythms when attention is directed to these rhythms [36], one would then expect those focused on their task to pay less attention to their synchrony relative to the other drummer, and hence to show less difference in reward related brain activity between *synch* and *asynch* conditions. The data support this hypothesis: the ease of rhythm acquisition before scanning predicted the magnitude of the activity difference between the *synch* and *asynch* conditions in the right caudate, a region that was sensitive to monetary reward (as demonstrated using the localizer task).

That those participants that learned to drum more easily have more activity in the caudate, a region associated with reward processing, has face validity when considering our experience of dancing, chanting or other synchronized activities: when we struggle to perform such a task, we tend to focus our attention inwards, on that task, and we shut out any distractors, including our social environment. Once we become more proficient, we open up, and start to enjoy synchronizing with others. It then becomes fun to dance, chant or drum in synchrony with others. Here, we propose that the neural correlate of this phenomenon may depend on caudate activity increasing with both synchrony and ease of performance. Because studying the effect of ease of acquisition was a secondary aim of our study, we did not prescreen participants to ensure a homogeneous distribution of participants over the range of ease. Accordingly, our results are strongly influenced by a small number of participants with extreme ease or unease of acquisition.

Two alternative explanations of this effect seem less likely. First, one could assume that the entire experiment became more rewarding for participants who acquired the rhythm more easily. However, there was no relation between caudate activity during asynchronous drumming and ease of rhythm acquisition, which argues against that assumption. Second, one might reverse the causality and propose that it is the higher activity in the caudate, a region known to play a role in synchronization or pulse-keeping, that causes some participants to be better drummers rather than the better drumming leading to more activation in the reward related caudate. This alternative account would predict a link between the proficiency of drumming during scanning and caudate activity – a link we failed to find.

Finally, our prosocial commitment test revealed that participants helped their last drum partner more if she had drummed in-synch with them several minutes ago in the manipulation run. These results are consistent with behavioral studies that demonstrate a link between synchronized musical activity and prosocial behavior [7], [8], [10]. In addition, the degree of activation in the right caudate while experiencing synchronous drumming predicted the number of pencils the individual would pick up to help the synchronous experimenter. This caudate activity occurred in a region that is, as demonstrated by our localizer experiment, responding to basic monetary rewards [16], [17], [18], [19], known to be essential for modulating prosocial behavior [21], [22] and necessary for reward-based decision making (i.e., modulation of a future decision based on the past experience of reward [24], [25], [26], [27], [28]). In the context of our results, this suggests that synchronized activity with a co-drummer activates reward signals in the caudate during drumming (in the scanner) and this reward history becomes associated with the synchronized co-drummer of the manipulation run. At a later point in time, when the synchronized experimenter dropped the pencils, this reward history increased the propensity of the participant to help that experimenter. This mechanism is compatible with the role the caudate plays in non-musical decision-making and reinforcement learning [24], [25], [26], [27], [28].

This link between the activity in a region associated with reward, the caudate in particular, and subsequent prosocial behavior could help us understand why musicians feel so rewarded and bonded after a successful jam session. Our choice of drumming in this experiment, as an example of synchronized activity, does not reflect a belief that there is something special about drumming. It was dictated by the practical consideration that drumming can be more easily performed in a scanner than rowing, dancing or marching together. We do believe, however, that similar neural processes apply to a range of other musical (e.g. chanting) and non-musical (e.g. rowing or marching) synchronized actions [37]. Here we investigated the simplest form of synchronization between participants: drumming the same notes at the same time. When an orchestra plays together, when two people tango or sing a duet, they coordinate the tempi of their actions at a higher, more abstract temporal level (e.g. the beat), and a sense of synchrony emerges even while taking turns, and when actions actually do not happen at the same time. Investigating whether the same neural signatures are triggered by such temporal coordination will be an interesting topic for further research.

Although previous behavioral studies had established the effect of synchronous activity on prosocial behavior, our results suggest that this may be true only for activities a particular individual masters easily: We found a marginally significant positive correlation between participants' ease of rhythm imitation and the prosocial commitment towards the synchronous co-drummer (see Fig. 1B, green line). There was no such correlation between ease of rhythm imitation and prosocial behavior for the co-drummer who was not in synchrony in the manipulation run (see Fig. 1B, red line). Similarly, the ease of rhythm imitation was significantly positively correlated with brain activity in the right caudate, which in turn predicted the number of pencils the participants picked up. As discussed in detail below, these effects depend on a small number of participants at the extreme of ease distribution – and need to be replicated with larger samples.

One might question whether the results we found might simply be due to fatigue: could participants that acquired the rhythm more easily have found the whole experiment more rewarding (hence more caudate) and less exhausting, leaving them more energy to help the experimenter? Two arguments speak against this interpretation. First, caudate activity did not increase with ease in general, but only during synchronized drumming. Second, there was no correlation between ease of rhythm imitation and prosocial behavior for the co-drummer who was not in synchrony in the manipulation run (see Fig. 1B, red line), showing that ease of rhythm imitation did not facilitate helping behavior in general.

This experiment is the first study showing how synchronized activity is linked to prosocial behavior in the brain. At the onset of this experiment, there was no indication of how strong the effect of synchrony on brain activity might be and there was no strong evidence that the effect of synchrony might be restricted to participants that master the task easily. Hence, we performed the study on a number of participants, 18, that is typical for neuroimaging studies. To maximize our potential to test the hypothesis that reward related regions of the caudate may play a key role, we followed a region of interest approach in this paper. This approach warrants moderate statistical thresholds (p<0.005), therefore providing the power to detect modest effect sizes with 18 participants while controlling the risk for false positives through a-priori hypotheses about location. However, it is difficult to trust activations outside of the region of interest with such thresholds, which explains why we do not interpret activations outside our ROI in this paper. With hind-sight of the fact that our study found an effect of ease of rhythm acquisition, 18 participants may have been too few: critical findings depend on a small number of participants that acquired the task easily. As a consequence, most of our results are at the edge of significance. We therefore recommend interpreting our results with care and seeing their foremost value in channeling and inspiring future research. Specifically we believe to afford the field experimental leverage on the relation between synchrony and prosocial behavior by providing new testable hypotheses: (a) the effect of synchronized actions on brain activation and prosocial behavior depends on participants that master the task well enough to have cognitive resources left to attend to the level of interpersonal synchrony, and (b) the effect of synchronized behavior on prosocial behavior is conveyed by reward sensitive areas. Our data are compatible with both hypotheses. However, for the data to provide strong evidence for these hypotheses, the effects in the present study are too close to significance levels and too often dependent on a small number of participants at the extremes for our variables of interest. For example, to ensure that correlations would not depend on a small number of individuals at the extremes of the ease continuum, future studies should preselect a sufficient number of participants at these extremes. The effect of synchronized activity itself would be better studied in a full group of participants preselected to master the task easily. This would provide more statistical power to compare brain activation during synchronous and asynchronous drumming and test the link between brain activity and prosocial behavior. Finally, experiments that compare musical and non-musical temporally coordinated actions would help clarifying whether our findings are limited to music.

Another question for future research might be to identify the nature of the psychological states that are related to our neural findings. Given the synchrony dependent activity in monetary reward regions we measured, one might wonder if reward in general or synchrony in particular may be key to the prosocial effect we measured. Future experiments could design activities not involving synchrony that would be more enjoyable with one co-player than with another to test whether such activities would have been equally effective at facilitating prosocial behaviour and whether they would have triggered similar neural correlates. Kirschner and Tomasello (2010) argue against this notion by showing that children engaging in a musical activity involving synchrony helped their co-musicians more but children engaging in a similar, but non-musical/non-synchronous game did not [10].

In conclusion, we provide preliminary neural evidence for how experiencing synchrony in joint drumming could be linked to increased prosocial behavior. Our data suggest that the caudate (which also responds to monetary reward) relates synchronized activity to basic reward processing in the brain, and that a history of such reward with a particular person influences future decisions to help that person. Additionally, we provide preliminary evidence that these effects depend on the individual being skilled in the synchronized activity. Finally, we expect that similar effects exist for other musical or non-musical group activities performed in temporal coordination (e.g., chanting, drumming, dancing, rowing, marching). We trust that our study will spark new research that will confirm these effects in larger samples and investigate the role played by other brain structures in linking synchrony to prosocial actions.

## Supporting Information

Figure S1 Experimental set-up and timeline and stimuli used in the fMRI experiments. (A) Timeline of the whole procedure including the training, the fMRI experiment and the prosocial commitment test; (B) Trial structure of the drumming task; (C) Trial structure of the reward localizer task.
(TIF)

Video S1 The demonstration video, which was used both in the training and the fMRI experiment (presented in the beginning of the each block in both training and fMRI blocks).
(AVI)

Listening Example S1 Sounds of the rhythm that would be produced by a participant (higher drum tones) together with the sounds that simulate a synchronous experimenter (lower drum tones).
(MP3)

Listening Example S2 Sounds of the rhythm that would be produced by a participant (higher drum tones) together with the sounds that simulate the asynchronous experimenter (i.e., prerecorded rhythm, lower drum tones).
(MP3)

Table S1 Description of the typical behaviors of the participants during training with respect to their ‘ease of learning’ ratings
(DOC)

Table S2 Debriefing questionnaire
(DOC)

Table S3 Abbreviations used in the paper together with their meanings.
(DOC)

Table S4 Mean and standard deviation of the trials with mistakes for *synch* and *asynch* conditions
(DOC)

Table S5 Mean and standard deviation of the asynchronies of the participant's button presses relative the demonstrated rhythm.
(DOC)

Supporting Information S1 Analysis of the role of the experimenter
(DOC)

Supporting Information S2 Further Discussion: Regression analysis: brains activity *asynch* vs. *synch* drumming and ease of rhythm imitation
(DOC)
