# Supplementary figures and images for: Synchronized Drumming Enhances Activity in the Caudate and Facilitates Prosocial Commitment - If the Rhythm Comes Easily
Source: PLoS One. 2011 Nov 16;6(11):e27272. doi: 10.1371/journal.pone.0027272 (PMC3217964; doi:10.1371/journal.pone.0027272)

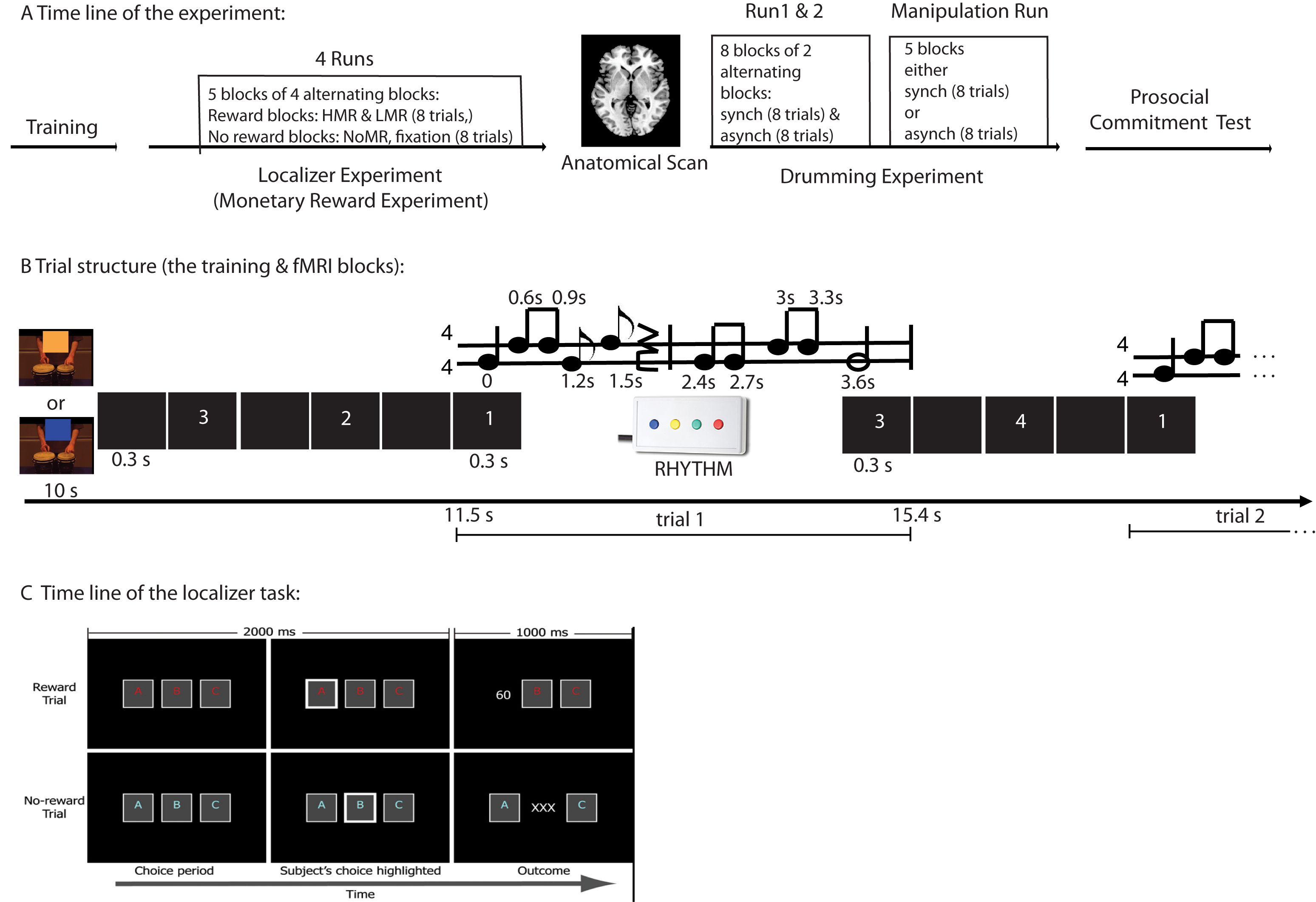

Supplement: Figure S1 — Experimental set-up and timeline and stimuli used in the fMRI experiments. (A) Timeline of the whole procedure including the training, the fMRI experiment and the prosocial commitment test; (B) Trial structure of the drumming task; (C) Trial structure of the reward localizer task. (TIF) [file pone.0027272.s001.tif]
