# Supplementary material for: Synchronized Drumming Enhances Activity in the Caudate and Facilitates Prosocial Commitment - If the Rhythm Comes Easily
Source: PLoS One. 2011 Nov 16;6(11):e27272. doi: 10.1371/journal.pone.0027272 (PMC3217964; doi:10.1371/journal.pone.0027272)
Supplement: Table S1 — Description of the typical behaviors of the participants during training with respect to their ‘ease of learning’ ratings (DOC) [file pone.0027272.s005.doc]

**Table S1.** Description of the typical behaviors of the participants during training with respect to their ‘ease of learning’ ratings

| **Ease** | **Description** |
| --- | --- |
| **1** | She watched the demonstration video 3 times. She could not reproduce the rhythm and asked the experimenter to show how to play the rhythm. |
| **2** | She watched the demonstration video 3 times. She was able to play the first 5 notes and missed the rest. She needed time until she was able to reproduce the rhythm correct. |
| **3** | She watched the demonstration video 2 times. Then she started reproduce the rhythm with some mistakes. |
| **4** | She was able to reproduce the rhythm after watching the demonstration video without mistakes. |
| **5** | She was immediately able to reproduce the rhythm after watching the demonstration video once. |
