# Supplementary material for: Synchronized Drumming Enhances Activity in the Caudate and Facilitates Prosocial Commitment - If the Rhythm Comes Easily
Source: PLoS One. 2011 Nov 16;6(11):e27272. doi: 10.1371/journal.pone.0027272 (PMC3217964; doi:10.1371/journal.pone.0027272)
Supplement: Table S2 — Debriefing questionnaire (DOC) [file pone.0027272.s006.doc]

**Table S2.** Debriefing questionnaire

|  | (very hard) |  |  |  | (very easy) |
| --- | --- | --- | --- | --- | --- |
| How easy was the rhythm of the experiment? | 5 | 4 | 3 | 2 | 1 |
|  |  |  |  |  |  |
| How much did you need to concentrate in order to play your drums? | 5 | 4 | 3 | 2 | 1 |
|  | (very much) |  |  |  | (very little) |
| How much did you like drumming with the person who was wearing a red t-shirt? | 5 | 4 | 3 | 2 | 1 |
|  |  |  |  |  |  |
| How much did you like drumming with the person who was wearing a blue t-shirt? | 5 | 4 | 3 | 2 | 1 |
|  |  |  |  |  |  |
| How much fun did you have while drumming with the person who was wearing a red t-shirt? | 5 | 4 | 3 | 2 | 1 |
|  |  |  |  |  |  |
| How much fun did you have while drumming with the person who was wearing a blue t-shirt? | 5 | 4 | 3 | 2 | 1 |
