# Supplementary material for: Synchronized Drumming Enhances Activity in the Caudate and Facilitates Prosocial Commitment - If the Rhythm Comes Easily
Source: PLoS One. 2011 Nov 16;6(11):e27272. doi: 10.1371/journal.pone.0027272 (PMC3217964; doi:10.1371/journal.pone.0027272)
Supplement: Table S3 — Abbreviations used in the paper together with their meanings. (DOC) [file pone.0027272.s007.doc]

**Table S3.** Abbreviations used in the paper together with their meanings.

|  | Ang Gyrs | angular gyrus |
| --- | --- | --- |
| * | BA44 | Brodmann area 44 |
|  | BOLD | blood-oxygen-level dependent |
|  | C.Vermis | cerebellar vermis |
|  | EPI | echo-planer imaging |
|  | fMRI | functional magnetic resonance imaging |
|  | GLM | general linear model |
|  | Hem | hemisphere |
| * | hIP1 | human intraparietal area 1 |
|  | IPL | inferior parietal lobule |
|  | MFG | middle frontal gyrus |
|  | MOG | middle occipital gyrus |
|  | midCingulateCrtx | middle cingulate cortex |
|  | MTG | middle temporal lobe |
|  | preCG | precentral gyrus |
|  | postCG | postcentral gyrus |
|  | SD | standard deviation |
|  | SPL | superior parietal lobule |
|  | supMGyrs | supramarginal gyrus |
|  | STG | superior temporal gyrus |
| * | TE 1.0 | primary auditory cortex |
| * | TE 1.1 | primary auditory cortex |
|  | Vox | voxel |

All brain areas were labeled using the Anatomy Toolbox for SPM (Eickhoff et al., 2005), with areas preceded by a ‘*’ based on probabilistic cytoarchitectonic maps.
