# Supplementary material for: Synchronized Drumming Enhances Activity in the Caudate and Facilitates Prosocial Commitment - If the Rhythm Comes Easily
Source: PLoS One. 2011 Nov 16;6(11):e27272. doi: 10.1371/journal.pone.0027272 (PMC3217964; doi:10.1371/journal.pone.0027272)
Supplement: Table S4 — Mean and standard deviation of the trials with mistakes for synch and asynch conditions (DOC) [file pone.0027272.s008.doc]

**Table S4**. Mean and standard deviation of the trials with mistakes for *synch* and *asynch* conditions

|  | Synch drumming | Asynch Drumming |
| --- | --- | --- |
| Missing a note | 0.44 ± 0.86 | 0.61 ± 0.92 |
| Stopping to play after several notes or skipping an entire trial | 0.50 ± 0.86 | 0.56 ± 0.78 |
| Playing the rhythm wrongly, mainly by playing the wrong notes | 1.17 ± 2.12 | 1.89 ± 2.19 |

Note: Means ± SD for the average number of trials with mistakes across participants are reported.
