# Supplementary material for: Synchronized Drumming Enhances Activity in the Caudate and Facilitates Prosocial Commitment - If the Rhythm Comes Easily
Source: PLoS One. 2011 Nov 16;6(11):e27272. doi: 10.1371/journal.pone.0027272 (PMC3217964; doi:10.1371/journal.pone.0027272)
Supplement: Table S5 — Mean and standard deviation of the asynchronies of the participant's button presses relative the demonstrated rhythm. (DOC) [file pone.0027272.s009.doc]

**Table S5.** Mean and standard deviation of the asynchronies of the participant’s button presses relative the demonstrated rhythm.

| MEAN | Note  2 | Note  3 | Note  4 | Note  5 | Note  6 * | Note  7 | Note  8 | Note 9 | Note  10 |
| --- | --- | --- | --- | --- | --- | --- | --- | --- | --- |
| synch (ms) | -68.3 | -12.2 | -17.1 | -3.4 | -291.2 | -2.3 | -11.5 | 15.3 | -15.7 |
| Asynch (ms) | -62.4 | -16.4 | -21.6 | 16.3 | -315.4 | -15.7 | 16.5 | -0.25 | 5.9 |
|  |  |  |  |  |  |  |  |  |  |
| SD |  |  |  |  |  |  |  |  |  |
| synch (ms) | 45.9 | 20.8 | 22.8 | 24.1 | 52.6 | 27.3 | 29.6 | 24 | 24.9 |
| Asynch (ms) | 67.2 | 30.1 | 34.3 | 47.7 | 77.6 | 33.0 | 33 | 43.8 | 40.3 |

Note: *6^th^ beat after the break is always played too early by the participants. Asynchronies are calculated relative to the preceding note, taking into account the duration of the requested rhythm (e.g.,[(onset beat3 – onset beat 2) – (requested duration, i.e. 300ms)].
