# Supplementary material for: Synchronized Drumming Enhances Activity in the Caudate and Facilitates Prosocial Commitment - If the Rhythm Comes Easily
Source: PLoS One. 2011 Nov 16;6(11):e27272. doi: 10.1371/journal.pone.0027272 (PMC3217964; doi:10.1371/journal.pone.0027272)
Supplement: Supporting Information S1 — Analysis of the role of the experimenter (DOC) [file pone.0027272.s010.doc]

**S1. Analysis of the role of the experimenter**

Given that during scanning the participants did not directly see with whom they were drumming, but only a color cue referring to the color of the t-shirt of the two experimenters, we explored if we had evidence that the participants associated this impoverished cue during scanning with a particular experimenter. We reasoned that such evidence would be present if at debriefing (see Table S2), the participant’s recollection of how much fun it was to drum with the two experimenters and how much they like the two experimenters correlated with the role played (*synch* or *asynch*) by the experimenter during the beginning of the experiment (including the training) and/or the manipulation run. For each participant, we gave an arbitrary binary score of one if the participant reported to have more fun with the experimenter associated with blue and zero otherwise, yielding a binary variable with one entry per participant. A separate variable contained the same for liking (i.e., 1 if participant liked drumming with the blue experimenter more, 0 otherwise). A third and fourth variable contained one if for that participant the blue experimenter played in-synch and a zero otherwise, for the beginning of the experiment and the manipulation run, respectively. Given that synchronous activity was supposed to increase social bonding, if participants correctly associated the color cues during the experiment with the experimenters, we expected the fun and liking variable to correlate positively with the variable encoding the role of the experimenters (color of the t-shirt) both before and during the manipulation run of the experiment. Our results support our assumption. We found positive correlations between fun and the role of the experimenter before (Pearson’s r = 0.64, p = 0.01) and during the manipulation run (Pearson’s r = 0.51, p < 0.05). Likewise, for liking before (Pearson’s r = 0.80 p < 0.001) and during the manipulation run (Pearson’s r = 0.66 p < 0.001). Moreover, when we compared these correlation coefficients, we found no significant difference between the role of the experimenter before the manipulation run and during the manipulation run for fun (t_(14)_ = 0.417, p = 0.34, one tailed) as well as for liking (t_(14)_ = 0.545, p = 0.29, one tailed).

Lastly, to ensure that the manipulation run had a contribution that was independent of the earlier part of the experiment, we used the residuals from a regression of the fun and liking scores and the role of the experimenter before the manipulation run and then correlated these residuals with the role of the experimenter during the manipulation run. This was significant for ‘residual liking’ (Pearson’s r = 0.71, p < 0.001) and ‘residual fun’ (Pearson’s r = 0.7, p < 0.001).
