# Supplementary material for: Synchronized Drumming Enhances Activity in the Caudate and Facilitates Prosocial Commitment - If the Rhythm Comes Easily
Source: PLoS One. 2011 Nov 16;6(11):e27272. doi: 10.1371/journal.pone.0027272 (PMC3217964; doi:10.1371/journal.pone.0027272)
Supplement: Supporting Information S2 — Further Discussion: Regression analysis: brains activity asynch vs. synch drumming and ease of rhythm imitation (DOC) [file pone.0027272.s011.doc]

**S2. Further Discussion: Regression analysis: brains activity asynch vs. synch drumming and ease of rhythm imitation**

The correlation between brain activation in the contrast *asynch*-*synch* in the right amygdala and the ease of rhythm imitation suggests that the participants who mastered the rhythm more easily activated the amygdala more strongly during asynchronous drumming. The amygdala has been implicated in the processing of neutral stimuli that are uncertain or ambiguous in nature, as well as stimuli that are difficult to predict in time (Bach, et al. 2008; Herry, et al. 2007; Hsu, et al. 2005). Thus, stronger activation in the amygdala during asynchronous drumming for participants who learned to drum the rhythm more easily could reflect the detection of random, and thus difficult to predict, deviations between the target rhythm and the notes of the experimenter. This finding, together with the finding on the caudate in the inverse correlation, suggest that those who acquired the rhythm more easily were relatively more sensitive to the difference between drumming with an in- or out-of-synch drum partner and found these two experiences rather different, with in-synch drumming leading to activity in reward sensitive areas of the caudate, and out-of-synch drumming leading to more amygdala activity.

**Literature:**

Bach, D.R., Schachinger, H., Neuhoff, J.G., Esposito, F., Di Salle, F., Lehmann, C., Herdener, M., Scheffler, K., and Seifritz, E. (2008). Rising sound intensity: an intrinsic warning cue activating the amygdala. Cereb Cortex *18*, 145-150.

Herry, C., Bach, D.R., Esposito, F., Di Salle, F., Perrig, W.J., Scheffler, K., Luthi, A., and Seifritz, E. (2007). Processing of temporal unpredictability in human and animal amygdala. J Neurosci *27*, 5958-5966.

Hsu, M., Bhatt, M., Adolphs, R., Tranel, D., and Camerer, C.F. (2005). Neural systems responding to degrees of uncertainty in human decision-making. Science *310*, 1680-1683.
